# Supplementary figures and images for: Methods optimization for the expression and purification of human calcium calmodulin-dependent protein kinase II alpha
Source: PLoS One. 2024 Jan 5;19(1):e0285651. doi: 10.1371/journal.pone.0285651 (PMC10769071; doi:10.1371/journal.pone.0285651)

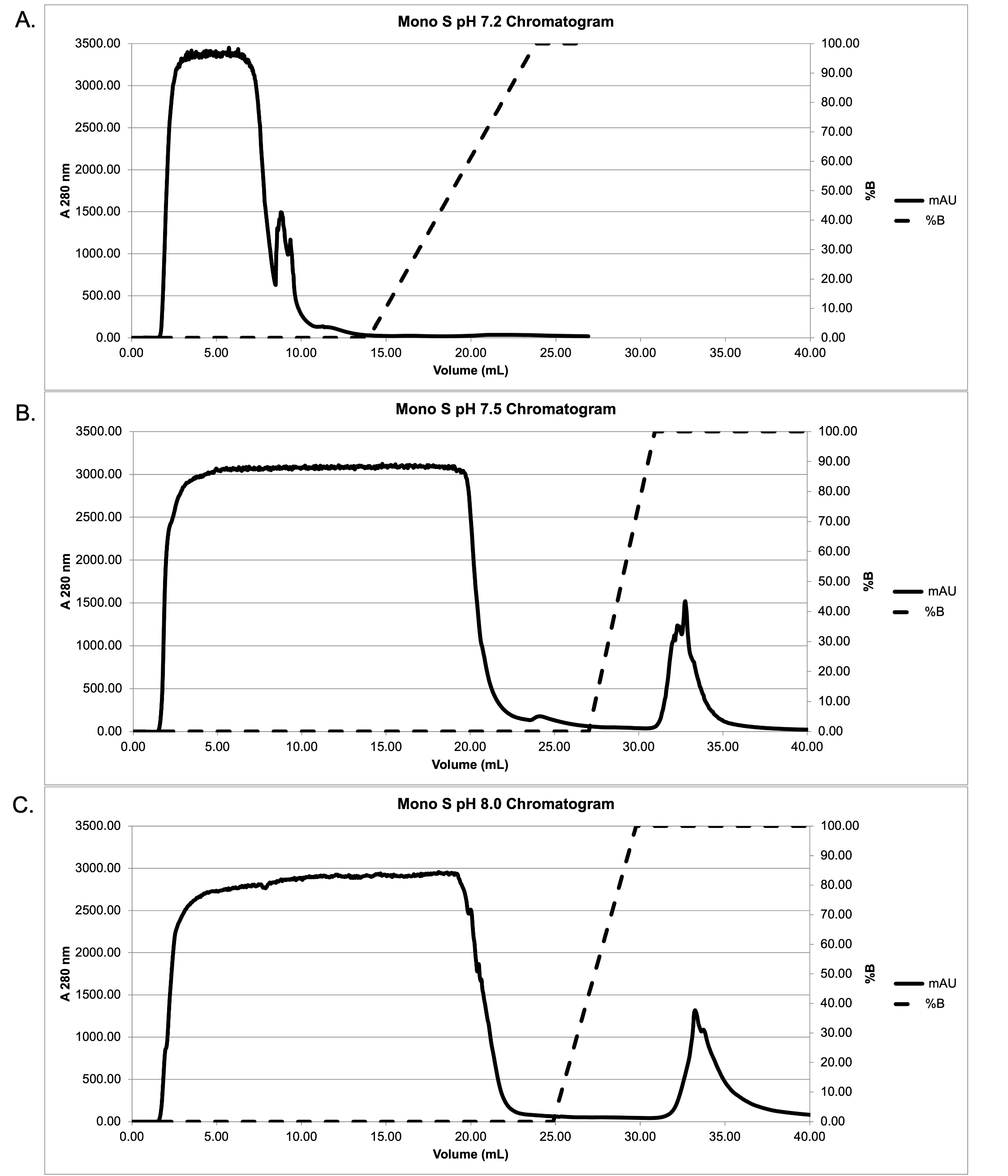

Supplement: S1 Fig — (A) pH 7.2, (B) pH 7.5, (C) pH 8.0. (TIF) [file pone.0285651.s001.tif]

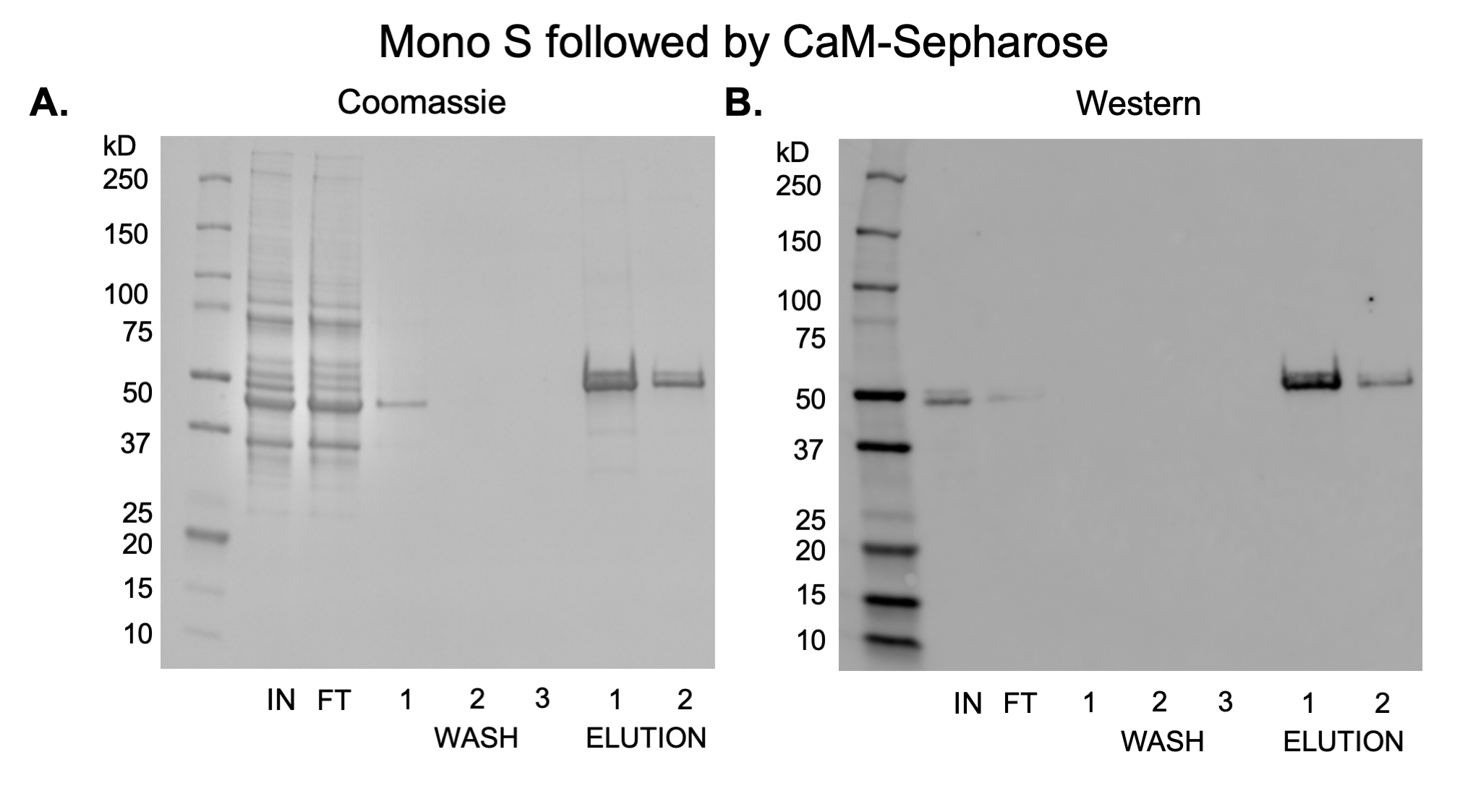

Supplement: S2 Fig — (A). SDS-PAGE followed by Coomassie stain, (B) Western blots stained with 6G9 anti-CaMKII primary antibody and detected with IRDye680RD secondary antibody. (TIF) [file pone.0285651.s002.tif]

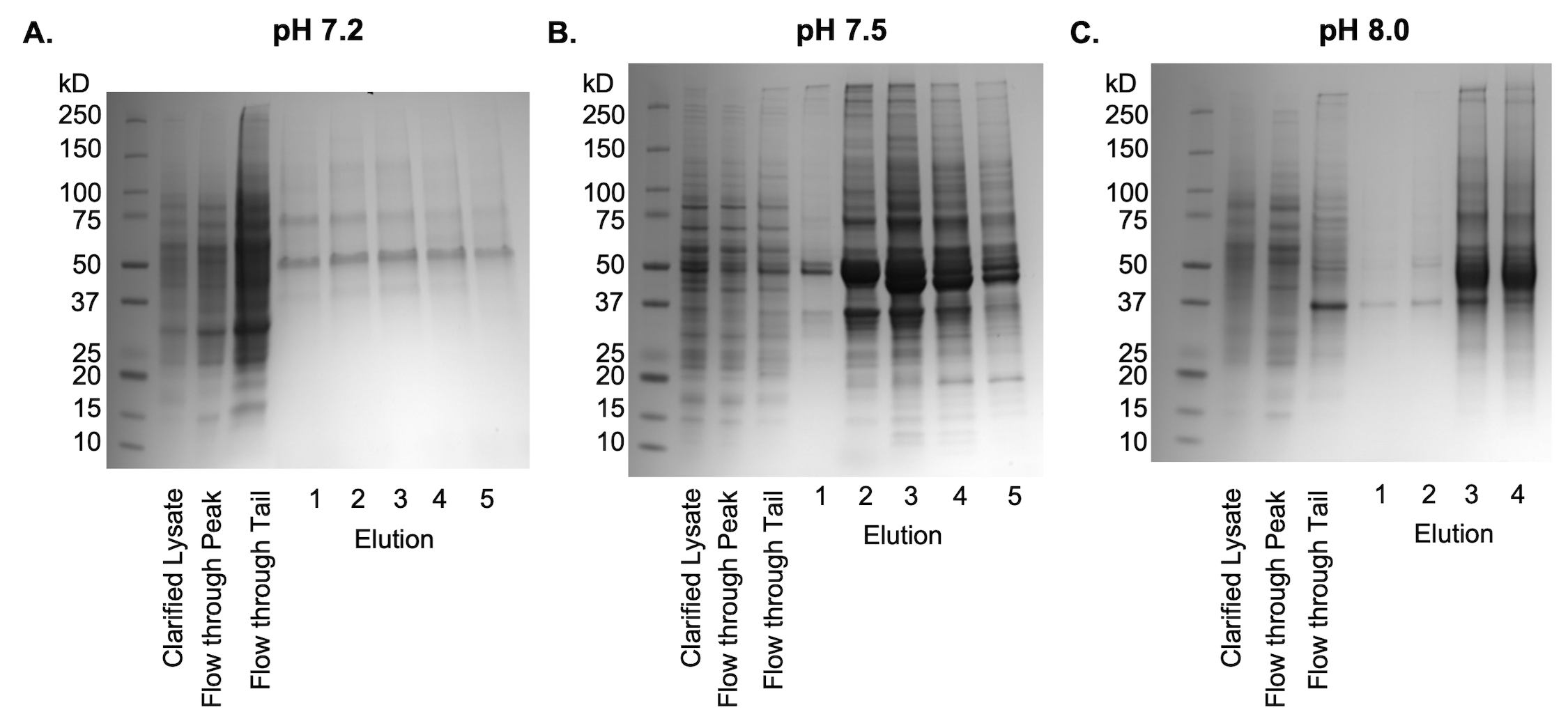

Supplement: S3 Fig — Initial separation of CaMKII from clarified lysate at (A) pH 7.2, (B) pH 7.5, and (C) pH 8.0. SDS-PAGE followed by Coomassie stain directly corresponds to the Western blots in Fig 3. (TIF) [file pone.0285651.s003.tif]

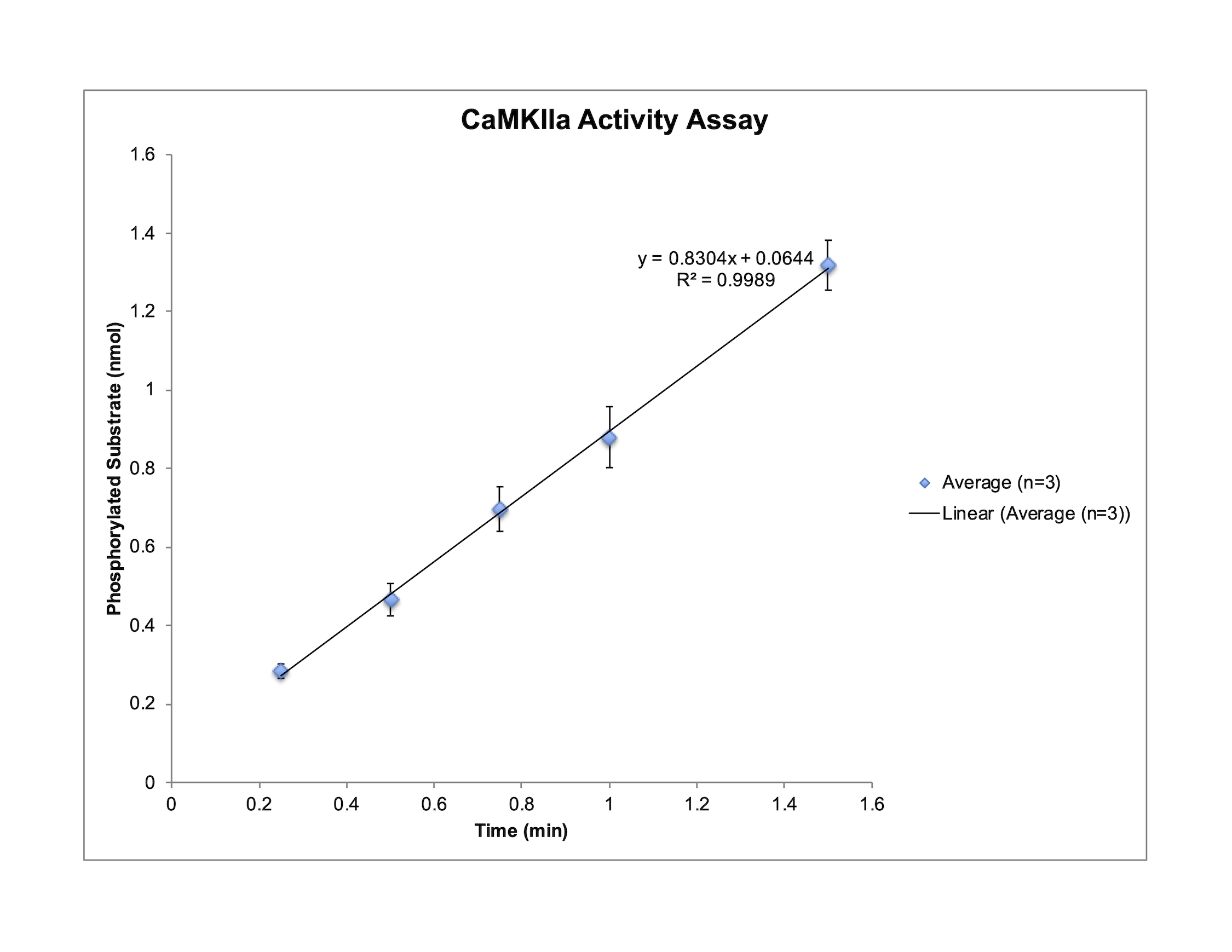

Supplement: S4 Fig — Radiolabeled ATP assay shows a linear response for at least 90 seconds. Specific activity is 5 μmol/min/mg. Error bars represent n = 3. (TIF) [file pone.0285651.s004.tif]
