# Supplementary material for: Methods optimization for the expression and purification of human calcium calmodulin-dependent protein kinase II alpha
Source: PLoS One. 2024 Jan 5;19(1):e0285651. doi: 10.1371/journal.pone.0285651 (PMC10769071; doi:10.1371/journal.pone.0285651)
Supplement: S1 Table — (DOCX) [file pone.0285651.s005.docx]

**S1 Table. Liquid chromatography mass spectrometry detection of CaMKIIα isoform B phosphorylation at Thr 286.**

| **Fragment Sequence** | **Number of Sites Phosphorylated** | |
| --- | --- | --- |
|  | 0 | 1 |
| **HPWISHRSTVASCMHRQETVDCLK** | 9 | 0 |
| **QETVDCLK** | 9 | 0 |
| **QETVDCLKK** | 11 | 0 |
| **QETVDCLKK** | 0 | 1 |
| **QETVDCLKKFNAR** | 5 | 0 |
| **STVASCMHRQETVDCLKK** | 1 | 0 |
| **Total Unmodified Counts** | 35 |  |
| **Total Phosphorylated Counts** | 1 |  |
| **Fraction Phosphorylated (%)** | 3 |  |
