# Supplementary material for: Methods optimization for the expression and purification of human calcium calmodulin-dependent protein kinase II alpha
Source: PLoS One. 2024 Jan 5;19(1):e0285651. doi: 10.1371/journal.pone.0285651 (PMC10769071; doi:10.1371/journal.pone.0285651)
Supplement: S2 Table — (DOCX) [file pone.0285651.s006.docx]

**S2 Table. Liquid chromatography mass spectrometry detection of CaMKIIα isoform B phosphorylation at Thr 305 / Thr 306.**

| **Fragment Sequence** | **Number of Sites Phosphorylated** | | |
| --- | --- | --- | --- |
|  | 0 | 1 | 2 |
| **GAILTTMLATR** | 98 | 0 | 0 |
| **GAILTTMLATR** | 0 | 2 | 0 |
| **GAILTTMLATRNFSGGK** | 1 | 0 | 0 |
| **KLKGAILTTMLATR** | 2 | 0 | 0 |
| **LKGAILTTMLATR** | 34 | 0 | 0 |
| **LKGAILTTMLATRNFSGGK** | 1 | 0 | 0 |
| **Total Unmodified Counts** | 136 |  |  |
| **Total Phosphorylated Counts** | 2 |  |  |
| **Fraction Phosphorylated (%)** | 1 |  |  |
