# Supplementary material for: Methods optimization for the expression and purification of human calcium calmodulin-dependent protein kinase II alpha
Source: PLoS One. 2024 Jan 5;19(1):e0285651. doi: 10.1371/journal.pone.0285651 (PMC10769071; doi:10.1371/journal.pone.0285651)
Supplement: S3 Table — (DOCX) [file pone.0285651.s007.docx]

**S3 Table. Liquid chromatography mass spectrometry detection of CaMKIIα isoform B phosphorylation at the nuclear localization sequence (Ser 332/333/334/335).**

| **Fragment Sequence** | **Number of Sites Phosphorylated** | | | | | |
| --- | --- | --- | --- | --- | --- | --- |
|  | 0 | 1 | 2 | 3 | 4 | 5 |
| **KSSSSVQLMESSESTNTTIEDEDTK** | 82 | 82 | 19 | 10 | 7 | 0 |
| **KSSSSVQLMESSESTNTTIEDEDTKVR** | 5 | 9 | 5 | 13 | 9 | 7 |
| **RKSSSSVQLMESSESTNTTIEDEDTK** | 0 | 16 | 14 | 6 | 5 | 0 |
| **SSSSVQLMESSESTNTTIEDEDTK** | 127 | 43 | 4 | 10 | 4 | 0 |
| **SSSSVQLMESSESTNTTIEDEDTKVR** | 57 | 15 | 3 | 15 | 11 | 0 |
| **Subtotal** | 271 | 165 | 45 | 54 | 36 | 7 |
| **Total Unmodified Counts** | 271 |  |  |  |  |  |
| **Total Phosphorylated Counts** | 307 |  |  |  |  |  |
| **Fraction Phosphorylated (%)** | 53 |  |  |  |  |  |
