# Supplementary material for: Methods optimization for the expression and purification of human calcium calmodulin-dependent protein kinase II alpha
Source: PLoS One. 2024 Jan 5;19(1):e0285651. doi: 10.1371/journal.pone.0285651 (PMC10769071; doi:10.1371/journal.pone.0285651)
Supplement: Attachment — Submitted filename: Response to Reviewers_v2.docx [file pone.0285651.s008.docx]

**PONE-D-23-12570: Methods Optimization for the Expression and Purification of Human Calcium Calmodulin-Dependent Protein Kinase II Alpha**

**Response to Reviewers**

*Reviewers comments are in italicized font.*

Reviewer #1:

*This paper compares two systems (insect cell vs bacteria) for the expression and purification of Human Calcium
Calmodulin-Dependent Protein Kinase II Alpha. The paper is generally very well presented in terms of readability, logical ordering, and presentation and discussion of data. I am sure it will be useful for others trying to express similar complex proteins. However, for it to be most useful to others, the following additional information should be supplied that relates to the baculovirus expression aspect:

(1) Source of Sf cells (line 91)*

Expression Systems has been added as the source on line 91.

*(2) Source of DH10Bac (line 86) and brief explanation of what these cells are or reference to a method for making recombinant baculoviruses using the 'Bac-to-Bac' method.*

Invitrogen has been added as the source on line 89.

A pFastBac vector (GenScript) incorporating the wild type human CaMKIIɑ isoform 2 gene at cloning site BamHI-Xhol was transformed into chemically competent *E. coli* DH10BaC cells (Invitrogen) that contain a bacmid shuttle vector and helper plasmid to facilitate the transposition of the gene into baculovirus DNA (PMID 21390847). Successful transformation was selected using TKG (Tetracycline, Kanamycin and Gentamicin) plates containing X-Gal (5-Bromo-4-Chloro-3-Indolyl β-D-Galactopyranoside, Invitrogen). White colonies were formed that indicated the disruption of the bacmid lacZα gene by successful transposition of the CaMKII gene from the donor pFastBac plasmid. This clarification has been added on line 87 - 93 in the Methods section.

*(3) The titre of the recombinant virus produced and the titre/MOI used to infect cells. The authors mention the importance of MOI (line 189) but do not mention when they describe infection details (lines 190-191 and in Fig 1). The infection details are too vague.*

The manuscript has been updated to include MOI values where relevant throughout the document including figure 2.

*(4) In Fig 1 Panel C there is no negative control for noninfected or mock-infected cells.*

This is true. Instead in Fig 1 at the first timepoint (36 hours post infection) we repeatedly found that very little protein was produced. Thus this is shown on the gel instead of the true negative control. The overall conclusion that we draw from the expression time course study remains the same.

Reviewer #2:

*The manuscript by Bolton et al. optimized an expression and purification method of Human CaMKIIα. Obtaining enough full-length protein is of great significance for the functional study of CaMKIIα, and this study provides a feasible solution. Therefore, I recommend publication in PlosONE after minor modifications.

(1) Line 99 and 219, I suggest converting the volume ratio to MOI (multiplicity of infection). For the baculovirus insect expression system, the MOI is a useful parameter, whereas the volume ratio does not provide sufficient information for the reader to repeat this study. For this, the titer of the P4 viral stock needs to be determined.*

The manuscript has been updated to include MOI values where relevant throughout the document including figure 2.

*(2 )Line 192 and 195, "post-infection" instead of "post-induction".*

The manuscript has been changed to “post-infection”.

Reviewer #3: Comments:

*The manuscript "Methods optimization for the Expression and Purification of Human Calcium Calmoudulin-Dependent Protein Kinase II Alpha" describes studies aimed at applying E. coli and baculovirus protein expression technology and accompanying purification schemes to develop an optimized process for obtaining high yields of CaMKIIalpha.

1. The authors did not quantify the amount of recombinant virus used in any of the studies. The authors use a percentage of virus for their studies. For completeness the authors should have quantified the virus titer of their P4 preparation.*

The manuscript has been updated to include MOI values where relevant throughout the document including figure 2.

*2. Why did the authors choose Tni vs. Sf9 cells for their 200 ml production runs?*

We chose Tni cells for protein expression because it had been recommended by the vendor (Expression Systems) for greater protein expression over Sf9/Sf21.

*3. The authors did not clearly state the advantages of their "optimized" methodology over that of the previously published studies using BEVS.*

Thank you for pointing this out. The previously published studies use parts of these methods in differing combinations. However, it is not clear from any single publication how to best select the parameters of expression system type, culture volume, pH, ion-exchange chromatography type, and/or affinity chromatography method in such a way that minimizes effort and handling time while demonstrating enriched yield of purified, mondisperse, oligomeric CaMKII. Our advantage is that we show reduced preparation and handling time by choosing the best parameters that minimize our volumes of culture and chromatographic columns. We do this by combining a smaller (200 mL) volume of baculovirus-insect culture with a pre-prepared, small 1 mL column volume Mono S ion-exchange chromatography method, targeted at pH 7.5, followed by batch-method Cam-Sepharose affinity chromatography. This has now ben added to the discussion.

*4. I did not see ref. 8-10 mentioned within the manuscript text. Ref 10 is cited in Table 1.*

References 8-10 were removed from an earlier text revision that included beta-CaMKII, The references accidentally remained in the bibliography (and Table 1) after editing. This has been fixed.

*5. The quantity of protein loaded onto gels was not stated.*

For protein purification gels, lanes were loaded with equal volumes (2μl for gels with cell lysate, and 5μl for gels with purification steps). The quantity of protein for each lane was not measured. This explanation has now been added to the methods section (line 148 – 149).

*6. Ref 2 is not in the text. I suspect it should be linked with ref. 1.*

Thank you for catching this. The references have been fixed.

*7. Ref 7- protein kinase II1 1. I believe 1 1 should be deleted.*

Thank you for catching this. The references have been fixed.
